# How can industrial intelligence change the employment structure of the floating population?

**DOI:** 10.1371/journal.pone.0297266

**Published:** 2024-05-06

**Authors:** Li Siyao, Wang Ya, Li Yingfeng

**Affiliations:** 1 School of Labor Economics, Capital University of Economics and Business, Beijing, China; 2 School of Tourism Management, South China Normal University, Guangzhou, China; Babes-Bolyai University, Cluj-Napoca, ROMANIA

## Abstract

As the wave of industrial intelligence (AI) swept, the demographic dividend era in the Chinese labor market continued to decrease. This study aimed to explore how AI reshaped the labor employment structure of the floating population. Additionally, it clarified the internal mechanism of AI on the employment structure of the floating population based on the existing AI model and the theoretical model of AI technology. At the same time, the workforce was divided into high-, medium-, and low-skilled groups according to education level. Empirical analysis was conducted using relevant data from 31 Chinese provinces spanning from 2012 to 2018. The aim was to test the impact of AI technology on the employment of different types of floating populations. The results indicated that: (1) industrial robots impacted heterogeneous skilled floating population labor by bipolar promotion and central substitution. (2) The application of industrial robots had a promotion effect on unfinished school and primary school groups, a substitution effect on middle school, high school/technical secondary school, and college specialties, and a promotion effect on college undergraduate and graduate students. (3) Distinguish employment status, industrial robot application had a significant negative impact on low-skilled employees and significant positive effects on high-skilled employers. Hence, it was recommended to put forward corresponding policy suggestions to address this issue.

## Introduction

As artificial intelligence (AI) sweeps the world, how technological progress changes the labor market is a hot issue for many scholars. Economist Keynes proposed the term "technical unemployment”. That is, the technological progress in the labor market accelerates and improves productivity, but with the replacement of machines. Studies have predicted that 400 to 800 million global jobs will be replaced by machines by the 1930s, and China will also have 31% of its working hours replaced by machines. Western countries, such as the United States, Europe, and other countries, have implemented the "industrial 4.0" development strategy, upgrading the transformation of industrial intelligence to reshuffle the industrial labor market. The labor employment structure of these countries has changed. The labor demand is reflected in not only quantity but also quality. AI has replaced jobs, and the number of high- and low-skilled jobs has increased. On the one hand, AI has received great importance in policymaking in China. In 2015, initiatives such as Made in China 2025 and the 19th National Congress emphasized the national strategy of intelligent manufacturing. By closely aligning with the changing times, the development of intelligent robots continued to inject impetus into the change in the Chinese labor market. On the other hand, since it is no longer the previous demographic dividend era, "labor shortage”, "recruitment”, structural supply and demand mismatch, and shortage of talent now exert a profound impact on the labor market structure.

The wave of industrial intelligence has accelerated the beneficial supplement of AI in the labor market, thus achieving efficient "machine substitution". It can effectively alleviate the "labor shortage" and "labor difficulties" in some labor markets. Moreover, the entry of machines into the labor market has also created several new industries and job opportunities, thereby increasing the labor demand for different skills. Of particular importance is the impact on the floating population, as changes in the labor market directly affect their willingness to settle and their choice of residence. In the context of China, the employment behavior of migrant population is unique. Generally speaking, they are at a disadvantage in the employment market and social welfare because of their low education level and professional skills, and lack of local hukou. The floating population is particularly vulnerable to the impact of AI, with a large number of low-skilled workers facing the risk of being replaced by intelligent machines. Based on the aforementioned considerations, the construction of a model and the quantitative study of the impact of industrial intelligence on the labor structure of the floating population have certain theoretical guiding significance for the policy formulation of government population and economic development strategies. Furthermore, it has practical implications for ensuring employment stability among the floating population in the destination region.

The innovation of this article is reflected in the following two aspects: First, this article focuses on the employment problem of China’s migrant population. In the context of China, migrant population does not have local household registration and is at a disadvantage in the labor market. Especially when AI enters the enterprise, it is very likely that enterprises will introduce more machines to replace some labor, most of which are migrant population. Through the discussion of this article, we can try to guide migrant population how to obtain more stable employment and alleviate the risk of being replaced by AI. Second, this article verifies the polarization effect of AI on labor force, proving that such effect is effective in the context of China’s migrant population. Both theory and empirical evidence prove that the development of AI brings creative effect to low-skilled and high-skilled labor force, and substitution effect to medium-skilled labor force. At the same time, the substitution effect for employees is stronger, and the creative effect for employers is stronger. The research suggestions of this article are to encourage migrant population to improve their ability to adapt to AI.

## Review of the literature

### Three effects of AI on the labor market

Digital technologies, closely related to artificial intelligence, have become an important driver of economic growth. It is not only a key direction for future sustainable development, but also has an important impact on the labor market [1,2]. Autor et al. explored the impact of AI on the labor market macroeconomic model and laid the analysis framework foundation for subsequent research [3]. The model for the "task-based model" explains the interaction effect of technology and labor, proving that technological progress on labor demand also has productivity and new employment effects. Acemoglu and Restrepo believed that the substitution effect would have an impact on the labor market [4,5]. Technological advances can bring a lot of industrial robots to replace the workforce in the labor market, thereby reducing costs while increasing productivity and thus potentially reducing the balanced wage level of labor. In terms of the productivity effect, Haltiwanger et al. identified three pathways. First, the large-scale use of AI automation brought about by technological advances in industrial enterprises would greatly save the production costs of enterprises. The larger the scale is, the lower the cost of the final product and services produced. The scale effect can encourage companies to further expand their operations, which in turn increases the demand for labor employment by increasing productivity. Second, the demand for products and services in related industries will also increase. Expanding the scale of related industries will also increase the labor demand for non-AI replacement jobs. Again, the scale effects will drive companies to scale up and increase the number of jobs while increasing labor productivity [6]. The bargaining power of the workforce will also improve, thus increasing the balanced wages in the labor market. In terms of the job creation effect brought by technology, macroeconomic models adopted by Acemoglu and Restrepo demonstrate that under the automation conditions of AI, new industries and services will accelerate in the economic market and produce new jobs [4,7].

### Changes in the labor market employment structure brought about by AI

Technological advancements (AI) not only contribute to economic growth and increased productivity but also have an impact on the labor market. Schumpeter’s theory of ’destructive creativity’ highlights the concept of creative destruction as a fundamental process in technological progress and economic development. This process involves the emergence of innovative ideas, processes, products, and organizations, while routine tasks are continuously phased out [8]. Autor et al. put forth theoretical models that highlight the impact of technological progress on different-skilled workforces, revealing a parretn of polarized employment [7,9]. Generally, the work content held by the skilled workforce is most easily replaced by AI because its work involves programming, formatting, and routine activities. In contrast, a low-skilled job, although simple in nature, often requires hands-on machine operation and similar tasks, which leads to increased labor demand rather than being impacted by it. Conversely, high-skilled labor involves complex tasks and requires more mental activities, making it less likely to be replaced by AI. In fact, the progress of AI further promotes the demand for highly skilled personnel.

### Quantitative research on the impact of AI on labor structure

Several studies have quantified the likelihood of occupational substitution due to AI, revealing varying impacts on different occupations. For instance, in the United States, approximately 47% of occupations are susceptible to substitution, while the figures are around 6% in Korea, 12% in Austria, 42% in Canada, and roughly 33% overall [10]. Additionally, many studies suggest that AI will also create new employment opportunities. With the rise in popularity of computers and the application of software technology in various industries, new job opportunities have emerged. These include roles in software and application development, database design and analysis, network security and maintenance, among others [11]. In addition to its destructive and creative effects, AI had led to a reshaping of work tasks. This has resulted in a structural restructuring of jobs, which has had a heterogeneous impact on labor with different skills. Scholars have referred to this phenomenon as ’Skilled-Biased Technological Changes’ (SBTC). The analysis of the empirical data of the manufacturing industries in European and American countries confirmed that AI has the strongest substitution effect on the medium-skilled labor force while positively affecting the high- and low-skilled labor forces [12,13]. Lv and Zhang conducted an in-depth analysis of China’s manufacturing industry and showed similar results, with an increased employment structure for high- and low-skilled workforce, an increased proportion of high- and low-technology industries, and a decreased proportion of employment in the technology industry [14]. Hao, using the education degree of labor to analyze the technical level of various industries, found that different industries exhibit an “N”-shaped employment and skills structure. Automation leads to a decline in employment for middle-skilled workers, while high- and low-skilled workforces demonstrate an increasing trend [15]. Qu and Cheng found that the employment structure of migrant workers, namely, the floating population, showed a polarization trend, and the growth rate of high- and low-skilled positions was higher than that of middle-skilled positions [16]. From the perspective of industrial structure transformation and upgrading and the open-trade economy, Li found that the polarization trend of China’s employment structure was verified again [17]. Sun and Hou further integrated industrial structure upgrading, regional differences, industrial intelligence, and employment structure, and a macroeconomic theoretical model was constructed to empirically analyze and demonstrate the polarization and internal mechanisms of labor employment [18].

In summary, foreign studies derived empirical data from European and American countries through macroeconomic theoretical models and reached a basic consensus on the trend of employment polarization. On the basis of the basic ideas and research framework, the existing research in China has used a series of Chinese data to verify the polarization. At present, the unified conclusion is that AI affects the employment structure of the workforce. Compared with the whole labor market, the labor market of the floating population is more sensitive. However, the related literature specifically focusing on the impact of AI on the employment structure of the floating population is limited. Furthermore, a standard theoretical model for analyzing this impact has yet to be established. Building upon this gap, this study proposes an analytical framework to examine the employment structure of the floating population in relation to AI. Drawing inspiration from influential works by foreign scholars Acemoglu and Autor [7] as well as domestic scholars such as Sun and Hou [19], this study developed a macroeconomic theory to derive insights and analyze the mechanisms underlying the impact of AI on the employment structure of the floating population. An empirical analysis is also conducted to explore its internal differences and far-reaching effects.

## Theoretical model derivation

### Equilibrium analysis of the basic mode

The manufacturer used the Z skill level in task i to construct the profit function. See Eq (1) for more details:

$$\prod_{Z}(i)=p(i)y(i)-\int_{0}^{A_{Z}}R_{z}(j)k_{Z}(i,j)dj-W_{Z}=\beta p{\left(i\right)}^{1/\beta }A_{Z}\alpha_{Z}(i)Z(i)-W_{Z}Z(i)$$ (1)

Assuming a fully competitive market, all interval tasks needed to be performed. To achieve balance, there is a low-skilled task IL when i <IL, and there is a high-skilled task IH when i >IL. That is, different skilled floating workforces undertake different tasks. The medium- and low-skilled workforce cannot perform the complex work of high-skilled labor, and the high-skilled workforce will not take up low-yield and low-skilled jobs. Hence, m(i) = h(i) = 0 when 0 < i < IL, when IL < i < IH, I(i) = h(i) = 0, and when IH < i < 1, I(i) = m(i) = 0.

### Equilibrium analysis of the AI input

The manufacturer invests AI capital to build the production function of task i. See Eq (2) for more details:

$$y(i)=p{\left(i\right)}^{(1-\beta )/\beta }[A_{L}\alpha_{L}(i)l(i)+A_{M}\alpha_{M}(i)m(i)+A_{H}\alpha_{H}(i)h(i)+A_{Q}\alpha_{Q}(i)q(i)]$$ (2)

where q(i) and Q(i) show the number and efficiency of AI capital in conventional production task i and unconventional production task αQ (i) = 0, respectively. A design was embodied in multiple Acemoglu classics and further categorized to represent the production function. See Eq (3) for more details:

$$\begin{array}{l} y(i)=p{\left(i\right)}^{(1-\beta )/\beta }A_{L}\alpha_{L}(i)l(i)\;0<i<I_{L}\\ y(i)=p{\left(i\right)}^{(1-\beta )/\beta }A_{M}\alpha_{M}(i)m(i)+A_{Q}\alpha_{Q}(i)q(i)\;I_{L}<i<I_{H}\\ y(i)=p{\left(i\right)}^{(1-\beta )/\beta }A_{H}\alpha_{H}(i)h(i)\;I_{H}<i<1 \end{array}$$ (3)

This classified production function is interpreted as the task interval of three different skilled floating population workforces. In (0, IL) range, for unconventional production tasks, but simple tasks, only manual labor can be completed by low-skilled floating population labor. In (IL, IH) range, for routine production tasks, task types are more complex, but a high degree of programming can be completed by the medium-skilled floating population workforce. In (IH, 1) interval, for unconventional production tasks, the tasks are complex, need high mental labor and technical requirements, and should be completed by the high-skilled floating population labor force.

AI capital is reflected in the routine task of the medium-skilled floating population workforce; therefore, the question is exactly how to replace it. The present study constructed a better idea of thought. Within the interval (IL, IH), a subinterval exists (I-ε, Iε). The productivity of AI capital is αQ(i). Corporate decision makers are more inclined to use AI capital to replace the medium-skilled floating population workforce, termed machine replacement. For the subinterval (I-ε, Iε), the productivity of AI capital αQ(i) is not higher than that of skilled migrant workers, and no substitution occurs. When 0 < i < IL`, m (i) = h(i) = q(i) = 0; when I-ε < i < Iε, l(i) = m(i) = h(i) = 0. For part of the interval (IL, IH), except for the subinterval (I-ε, Iε), IL `< i < I-ε and Iε < i < IH`, l (i) = h (i) = q (i) = 0; when IH`< i < 1, l(i) = m(i) = q(i) = 0.

Under the constraints of minimizing production costs and maximizing production efficiency, AI capital will replace the medium-skilled floating population workforce in the corresponding range. The demand function of labor can be represented by the following equation. See Eq (4) for more details:

$$\begin{array}{l} l(i)=Y/[p{\left(i\right)}^{1/\beta }\alpha_{L}(i)A_{L}]\;0<I<I_{L}^{`}\\ m(i)=Y/[p{\left(i\right)}^{1/\beta }\alpha_{M}(i)A_{M}]\;I_{L}^{`}<i<I_{-\varepsilon },I_{\varepsilon }<i<I_{H}^{`}\\ h(i)=Y/[p{\left(i\right)}^{1/\beta }\alpha_{L}(i)A_{L}]\;I_{H}^{`}<i<1 \end{array}$$ (4)

Given the equal marginal productivity of the tasks performed by the same skilled migrant workforce, see Eq (5) for more details:

$$p{\left(i\right)}^{1/\beta }\alpha_{L}(i)=p{\left(i^{`}\right)}^{1/\beta }\alpha_{L}(i^{`}),i,i^{`}\in (0,I_{L}^{`})$$ (5)

Let P be the price index. PL, PM, and PH are the price indexes of unit tasks of low-, medium-, and high-skilled floating populations. See Eq (6) for more details:

$$\begin{array}{l} P_{L}=p{\left(i\right)}^{1/\beta }\alpha_{L}(i)=p{\left(i^{`}\right)}^{1/\beta }\alpha_{L}(i^{`})\;0<i,i^{`}<I_{L}^{`}\\ P_{M}=p{\left(i\right)}^{1/\beta }\alpha_{M}(i)=p{\left(i^{`}\right)}^{1/\beta }\alpha_{M}(i^{`})\;I_{L}^{`}<i,i^{`}<I_{-\varepsilon },I_{-\varepsilon }<i,i^{`}<I_{H}^{`}\\ P_{H}=p{\left(i\right)}^{1/\beta }\alpha_{H}(i)=p{\left(i^{`}\right)}^{1/\beta }\alpha_{H}(i^{`})\;I_{H}^{`}<i,i^{`}<1 \end{array}$$ (6)

The formula is added to obtain three types of skills of the floating population. For the total demand function of the workforce, see Eq (7) for more details:

$$\begin{array}{l} L^{d}=YI_{L}^{`}/(P_{L}A_{L})\\ M^{d}=Y(I_{H}^{`}-I_{L}^{`}-\varepsilon )/(P_{M}A_{M})\\ H^{d}=Y(1-I_{H}^{`})/(P_{H}A_{H}) \end{array}$$ (7)

At this point, a slight change in form can yield the relative needs of the three types of skilled floating labor. See Eq (8) for more details:

$$\begin{array}{l} \frac{M^{d}}{L^{d}}=\frac{P_{M}A_{M}}{P_{L}A_{L}}\frac{I_{H}^{`}-I_{L}^{`}-\varepsilon }{I_{L}^{`}}\\ \frac{M^{d}}{H^{d}}=\frac{P_{M}A_{M}}{P_{H}A_{H}}\frac{I_{H}^{`}-I_{L}^{`}-\varepsilon }{1-I_{H}^{`}} \end{array}$$ (8)

AI and floating population labor substitution are related to the subrange of AI ε. The related research data show that the continuous development of AI ensures the goal of minimizing production costs, thus achieving the automation of conventional production tasks. That is, the scope of ε is extended, implying that the demand for a middle-skilled floating population workforce decreases compared with that for a high-skilled and low-skilled floating population workforce. See Eq (9) for more details:

$$\begin{array}{l} \frac{d\ln(M^{d}/H^{d})}{d\varepsilon }=\frac{-\beta_{H}^{`}(I_{H}^{`})\beta_{L}^{`}(I_{L}^{`})+\beta_{H}^{`}(I_{H}^{`})/I_{L}^{`}}{(I_{H}^{`}-I_{L}^{`}-\varepsilon )/\Delta }<0\\ \frac{d\ln(M^{d}/L^{d})}{d\varepsilon }=\frac{-\beta_{H}^{`}(I_{H}^{`})\beta_{L}^{`}(I_{L}^{`})+\beta_{H}^{`}(I_{H}^{`})/(1-I_{L}^{`})}{(I_{H}^{`}-I_{L}^{`}-\varepsilon )/\Delta }<0\\ \beta_{H}(I_{H}^{`})=\ln\alpha_{M}(I_{H}^{`})-\ln\alpha_{H}(I_{H}^{`})\\ \beta_{L}(I_{L}^{`})=\ln\alpha_{L}(I_{L}^{`})-\ln\alpha_{M}(I_{L}^{`})\\ \Delta =[\beta_{H}^{`}(I_{H}^{`})-1/(1-I_{L}^{`})][\beta_{L}^{`}(I_{L}^{`})-1/I_{L}^{`}]+[1/(1-I_{H}^{`})\\ +1/I_{L}^{`}-\beta_{H}^{`}(I_{H}^{`})-\beta_{L}^{`}(I_{L}^{`})]/(I_{H}^{`}-I_{L}^{`}-\varepsilon ) \end{array}$$ (9)

Through a series of derivations and evolutions, we drew the following conclusions.

After theoretical derivation, it can be found that in both Western countries and China, with the development of artificial intelligence technology, automation will gradually replace a greater number of jobs held by a medium-skilled workforce. However, the impact on the replacement of highly skilled and low-skilled floating workforces within the floating population is relatively minimal. It is expected that the positive productivity effect and the employment increase effect may gradually increase.

Based on this, this study proposed two research hypotheses.

Hypothesis 1: The application of industrial robots replaces the employment of the current floating population workforce.
Hypothesis 2: The application of industrial robots has the effect of medium-skilled substitution and low- and high-skilled promotion in different skill employment groups. That is, the impact of industrial robots on a heterogeneous skill workforce was bipolar promotion and central substitution.

## Data, variables, and research design

This study used the data of industrial robots in China’s manufacturing industry, combined with the changes in industry employment and wage level, to empirically test the impact of industrial robot use on the labor market of the Chinese floating population and discuss the time trend and industry heterogeneity. The innovation of this study was from the perspective of the Chinese floating population. The literature primarily focuses on the United States, Germany, and other developed countries to examine the influence of industrial robots on a rapidly developing country in terms of manufacturing. Very few studies have focused on robot use and its influence in developing countries. Meanwhile, this study also sought instrumental variables based on the actual situation in China. We attempted to solve the possible endogeneity problems in the empirical model. Furthermore, this study examined the impact of industrial robot use on the number of jobs in different skilled workers. The findings of this study might provide a more comprehensive and profound understanding of the current labor market affected by "machine replacement" and formulate corresponding policies and measures to better promote new technologies such as AI to "escort" in China and promote the stable and healthy development of China’s economy.

### Model setting

The labor equilibrium model in the AI stage was constructed, which provided theoretical support for the competition equilibrium of different skilled workforces under the automation perspective. This study established the following empirical model of benchmark regression. See Eq (10) for more details:

$${floatingemp}_{jt}^{1,2...n}=\alpha_{0}+\alpha_{1}{PIR\;exposure\;to\;robots}_{jt}+\alpha_{2}X_{jt}+\delta_{1j}+\lambda_{1t}+\varepsilon_{1jt}$$ (10)

The aforementioned equation shows the effect of industrial robot permeability on the employment or compensation of the floating population. The dependent variable upper target 1,2…n represents the floating population of different employment identities and skill categories; j and t of each variable represent the province and year, respectively; α and β represent the coefficients; α1 and β1 are the coefficients of the core explanatory variable, indicating the marginal substitution effect of industrial robot permeability on the employment demand of the floating population; X represents a series of control variables; δ and λ represent the provincial fixed effect and year fixed effect, respectively; and ε indicates the error item.

### Internal issues

The core independent variable, industrial robot permeability, affected the dependent variables of floating population employment demand and salary. Enterprises’ decisions to introduce industrial robots are often driven by the prevailing "labor shortage" and "talent shortage" challenges in recent years as well as the need to address labor supply and demand contradiction and rising labor costs [19]. Therefore, endogeneity problems might exist in core independent variables that need to be solved using exogenous instrumental variables. This study drew lessons from the findings of Acemoglu and Restrepo and proposed the use of American industrial robot data as a instrumental variable for Chinese industrial robot permeability, thereby mitigating potential endogeneity problems.

### Variable description and data sources

The time span of this study was 2012–2018, mainly considering two points. One was only the installation and stock of Chinese industrial robots after 2012. The Global Robot 2019 released by the International Federation of Robotics (IFR) noted that by 2018, the annual sales of China’s industrial robot market ranked first in the world for the sixth consecutive year. In China, the prosperity of the industrial robot market inevitably had an impact on the employment market. Second, after 2012, the labor structure faced the impact of the "superposition effect" of the third phase of population policy with the growth of the working-age population [20]. The secondary industry represented by the manufacturing industry also faced the dilemma of "labor shortages" and "talent shortages”, and the contradiction between supply and demand between the workforce and employment gradually became prominent. The floating population is the main force of the employment market, and the impact was more significant. We should consider how robots affect the employment effect of the floating population, what types of people face "machine replacement", and what the characteristics of "human–computer cooperation" are to promote the organic integration of AI and the employment market. Further research is needed to provide a more accurate and reliable basis for policymaking.

The main data sources of this study are three: (1) Industrial robot data. It came from the installation volume and stock data of industrial robots in the IFR report. To date, the IFR data included industrial robot data from 50 countries during 1993–2019. This study selected four types of industrial robot data as the research objects from the mining industry, manufacturing industry, power, heat, gas, and water production and supply industry, and construction industry during 2012–2018. (2) Floating population data. It came from the China Migrants Dynamic Survey (CMDS) during 2012–2018. These surveys have been carried out annually since 2009; they cover 31 provinces/ autonomous regions/ municipalities (hereinafter referred to as “provinces”). Data was released most recently in 2018, which is used in this paper. The CMDS sample is composed of floating population members aged 15 and above who have lived in their inflow locales for more than one month but do not have household registration (hukou) in those locales. Considering the industry characteristics of the employed floating population in the sample, the floating population in secondary industries such as mining, manufacturing, coal water thermal production and supply industry, and construction was selected as the research object. (3) Statistical Yearbook data. Variable data involving enterprises and regional levels were derived from the National Statistical Yearbook, China Labor Statistical Yearbook, China Industrial Statistical Yearbook, China Science and Technology Statistical Yearbook, and so forth. The specific index selection instructions were as follows. The statistical Yearbook data of China reflects the social and economic characteristics of recent years, and is an effective supplement to the study of this paper.

Explain variable $floatingemp_{jt}^{1,2\ldots n}$: This indicates the employment of the floating population in the secondary industry representing different employment identities and skill categories. In the regression, first, the employment demand and salary effect of the total floating population were measured. Second, employment identity was subdivided, and the employment demand and salary effect of employees, employers, and proprietary workers were measured. Finally, the skill categories were subdivided; education was the most common division. Based on the empirical results of junior high school and below set as low skills, high school/technical secondary school education as medium skills, and a higher degree or above as high skills, the employers and proprietary workers were divided into high-, middle-, and low-skilled categories according to employment status to further measure the effect of independent variables on floating population workforcess with different skill statuses. At the same time, considering the sample size of the sample data, the employment proportion of various floating populations was selected for a comparative analysis to ensure the effectiveness of the results.

Core independent variable ${PIR\;exposure\;to\;robots}_{jt}$: This indicates the penetration of the industrial robot, that is, the technical impact intensity induced by the robot. The industrial robot stock data of the IFR were mainly used, but these data only reported the robot stock at the national level of subindustries, with no data from subdivided units. Previous scholars have also noted this point. The approach was to measure the robot data at the provincial level [21,22] That is, the industrial employment structure of each province was adopted to form the initial weight and multiplied by the industrial robot penetration (coverage) of employees at the industry level. Hence, the penetration index of the industrial robot at the provincial level was determined. The specific calculation method was as follows:

First, the industrial robot permeability index at the industry level was calculated and recorded as IRit. See Eq (11) for more details:

$${IR}_{it}=\frac{{MR}_{it}}{{Labor}_{i,2010}}$$ (11)

This metric (IRit) measures the industrial robot penetration of t years in industry i. MRit represents the i industry t annual industrial robot stock, and Labori,2010 indicates the number of employees i in 2010 (base).

Second, the permeability index of the interprovincial industrial robot was estimated and recorded as PIR exposure to robotsjt. See Eq (12) for more details:

$${PIR\;exposure\;to\;robots}_{jt}=\sum_{i}^{n}\frac{{Emp}_{i,j,2011}}{\sum_{i}{Emp}_{i,j,2011}}\times \frac{{MR}_{it}}{{Labor}_{i,2010}}$$ (12)

This metric (PIR exposure to robotsjt) measured the number of industrial robots used per 10,000 people at the interprovincial level in different years. $\frac{{Emp}_{i,j,2011}}{\sum_{i}{Emp}_{i,j,2011}}$ is the proportion of urban employment in industry i in province j in 2011 to the total urban employment in 2011.

As shown by the aforementioned two types, the penetration of industrial robots at the interprovincial level was the weighted average of the use of robots at the industry level, and the weight was the share of the total employment in the industrial industry in the year before the inspection sample (2011). In theory, if an industry occupies a large share of employment in the region, then the robot growth in the industry has a greater impact on the labor market changes in the region, which directly affects the floating population employment market in the industry. The difference, on the one hand, comes from the difference in robot growth at different industrial industry levels between 2012 and 2018; on the other hand, it comes from the differences in industrial employment structure in different provinces.

Control variables: They mainly control the characteristic variables at the industrial industry and provincial levels. Referring to the study by Cheng et al., the industrial variables include the following: scale characteristics, measured by the average number of employees of industrial enterprises above the designated scale; capital investment, measured by the fixed asset investment of enterprises above the designated scale; and profit level, measured by the total profits of enterprises above the designated scale [23]. Considering the characteristics of the floating population and referring to Han and Zhao and Wei et al., the provincial level included the following: the urbanization rate, measured by the proportion of the urban population in each province; and the housing price level, measured by the average sales price of commercial housing in each province [24,25].

The description analysis of the various variables is shown in Table 1.

**Table 1:** Description analysis of the main variables.

Variable type	Variable name (unit)	Mean	Standard error	Minimum value	Maximum value
Construed variables	Total employment (one thousand)	2.025	4.498	0.017	21.024
	Number of employees (one thousand)	1.803	4.087	0.012	18.749
	Number of employers (a thousand)	0.068	0.155	0.001	1.306
	Number of proprietary individuals (a thousand)	0.137	0.252	0.001	1.903
	Number of high-skilled employees (a thousand)	0.202	0.400	0.000	2.877
	Number of medium-skilled employees (thousand)	0.355	0.859	0.001	5.076
	Number of low-skilled employees (a thousand)	1.247	2.980	0.008	15.793
	Number of high-skilled employers (a thousand)	0.010	0.024	0.000	0.206
	Number of medium-skilled employers (thousand)	0.018	0.051	0.000	0.539
	Number of low-skilled employers (a thousand)	0.041	0.090	0.000	0.562
	Average compensation (1000 yuan)	2.759	0.429	2.075	4.819
	Average employee pay (1000 yuan)	2.568	0.379	1.883	4.672
	Average employer compensation (thousand thousand)	5.654	2.341	-2.931	19.590
	Self-average compensation (thousand yuan)	3.154	0.584	1.902	5.138
Core explanatory variables	China’s industrial robot penetration (Taiwan/Wan people)	37.250	25.859	5.232	117.936
Instrumental variables	American industrial robot penetration (Taiwan/10,000 people)	35.668	12.117	11.961	71.686
Control variable	Average number of workers in industrial enterprises above designated size (take log)	5.080	1.351	0.579	7.293
	Investment in fixed assets of enterprises above designated size (Take logarithmic)	8.685	0.907	5.364	10.250
	Total profit of enterprises above designated size (take log)	6.729	1.449	1.503	9.193
	Urbanization rate (%)	56.699	13.006	22.750	89.600
	Home price level (Take the log)	8.510	0.444	7.919	10.026

## Analysis of the empirical results

### Influence of the application of industrial robots on the employment of the floating population in secondary industry

Table 2 reports the impact of industrial robot applications on the employment of the floating population in the secondary industry. The application of industrial robots had a significant negative impact on the employment of the floating population in the secondary industry. Model (1a) and model (1b) used simple benchmark regression, and model (2a) and model (2b) used instrumental variable regression. The two regression results were generally similar in the direction of action. To avoid the bias in the estimated coefficients caused by endogeneity problems such as omitted variables and reverse causality in model construction, this article uses instrumental variable regression to verify the results of the benchmark regression. In the selection of instrumental variables, it is important to ensure that the selected instrumental variables are both related to the core explanatory variable and do not affect the explained variable. The instrumental variable test results, LM statistics (35.912), and F statistics (1184.739) were significant at the 1% level, which justified the selection of instrumental variables. Controlling for various variables and effects, model (2a) showed that for each unit increase in industrial robot permeability, the total floating population employment of the secondary industry decreased by 0.029 units. Model (2b) showed that for each unit increase in industrial robot permeability, the employment proportion of the floating population in the secondary industry declined by 0.086 units. Both absolute and relative quantities indicated that the application of industrial robots had a substitution effect on the employment of the current floating population in the secondary industry. The robustness checks (Adjust the sample range, the samples of Beijing and Shanghai were excluded. And replace the measurement method) also yielded the same results. Thus validating hypothesis 1.

**Table 2:** Influence of industrial robot application on the employment of the floating population in the secondary industry.

model	Benchmark regression		Instrumental variable regression		robustness checks	
	(1a)	(1b)	(2a)	(2b)	Adjust the sample range	Replace the measurement method
	Total employment	Proportion of employment	Total employment	Proportion of employment	Total employment	Proportion of employment
Industrial robot permeability	–0.035*** (–3.490)	–0.096** (–2.249)	–0.029*** (–2.836)	–0.086** (–2.309)	–0.047*** (–3.565)	–0.088** (–2.235)
Constant terms	3.5474 (0.342)	36.430 (0.817)	1.270 (0.174)	32.422 (0.722)	3.6243 (0.388)	35.855 (0.866)
Control variable	Yes	Yes	Yes	Yes	Yes	Yes
Fixed effect of province and year	Yes	Yes	Yes	Yes	Yes	Yes
N	217	217	217	217	203	217
R2	0.982	0.954			0.977	0.949
LM			35.912***			
C-D Wald F statistic			1184.739***			

Note: *, * *, * * * are significant at 10%, 5%, and 1% significance levels, respectively; t statistics are reported in parentheses; instrumental variable regression standard error is robust standard error; given R2 significance in instrumental variable regression, the centered R2 and uncentered R2 results in instrumental variable regression are not reported here.

### Influence of industrial robot application on the employment of different employment identities

Table 3 reports the impact of industrial robot applications based on instrumental variable regression models on the employment of the floating population with different employment identities. Industrial robot application had a significant negative impact on employees, a significant positive impact on employers, and a positive but not significant impact on self-employed workers. Model (3a) and model (3b) showed that a 1-unit increase in industrial robot permeability resulted in a decrease of 0.320 units in employee employment and a decrease of 0.115 percentage points in the employee employment ratio. Model (3c) and model (3d) indicated that a 1-unit increase led to a 0.002 unit increase in employer employment and a 0.047 percentage point increase in the employer employment ratio. Model (3e) and model (3f) showed that the industrial robot permeability on proprietary worker employment ratio and employment was not significant. Both absolute and relative amounts showed that the application of industrial robots had a substitution effect on the current employment of employees, a promotion effect on employer employment, and no significant impact on the employment of self-employed workers.

**Table 3:** Effect of industrial robot application on the employment of the floating population with different employment identities.

Explain variables	Employees		Employer		Self-run	
	(3a)	(3b)	(3c)	(3d)	(3e)	(3f)
	Total employment	Proportion of employment	Total employment	Proportion of employment	Total employment	Proportion of employment
Robot	–0.320*** (–4.330)	–0.115** (–2.038)	0.002* (1.893)	0.047*** (3.242)	0.001 (0.247)	0.080 (1.611)
Constant terms	2.218 (0.331)	321.891*** (3.952)	–0.427 (–1.001)	25.229 (1.311)	–0.921 (–0.939)	–228.491 (–3.076)
Control variable	Yes	Yes	Yes	Yes	Yes	Yes
Fixed effect of province and year	Yes	Yes	Yes	Yes	Yes	Yes
Sample size	217	217	217	217	217	217

LM: 35.912***.

C-D Wald F statistic: 1184.739***.

### Influence of the application of industrial robots on the employment of different skills

Before dividing the floating population with different skills by education as the standard, least squares regression analysis was performed for each education group, and the skills were grouped according to the outcome characteristics. Tables 4 and 5 report the impact of industrial robot applications on the employment of floating populations to different degrees. As a result, the application of industrial robot employment had a significant positive impact on the total employment of unemployed, undergraduate, and graduate students and on the total employment of junior and high school groups. Furthermore, industrial robot application had a significant positive impact on the employment proportion of primary school groups and on the employment proportion of college, undergraduate, and graduate students.

**Table 4:** Influence of industrial robot application on the employment of the floating population in high schools and below.

Explain variables	I have not been to school		Primary school		Junior high school		High school/technical secondary school	
	(4a)	(4b)	(4c)	(4d)	(4e)	(4f)	(4g)	(4h)
	Total employment	The proportion of employment	Total employment	The proportion of employment	Total employment	The proportion of employment	Total employment	The proportion of employment
robot	0.001* (1.896)	0.009 (0.648)	0.003 (0.886)	0.136** (2.042)	-0.042*** (-4.242)	0.057 (1.194)	-0.012** (-2.503)	-0.064 (-1.355)
Constant terms	-0.088 (-0.189)	-34.563 (-1.553)	-4.343* (-1.857)	-109.771 (-1.842)	6.001 (0.593)	91.035* (1.692)	4.917 (1.548)	45.741 (0.942)
Control variable	Yes	Yes	Yes	Yes	Yes	Yes	Yes	Yes
Fixed effect of province and year	Yes	Yes	Yes	Yes	Yes	Yes	Yes	Yes
Sample size	217	217	217	217	217	217	217	217

**Table 5:** Influence of industrial robot application on the employment of the floating population in universities or above.

Explain variables	University junior college		University undergraduate course		Graduate student	
	(4i)	(4j)	(4k)	(4l)	(4m)	(4n)
	Total employment	Proportion of employment	Total employment	Proportion of employment	Total employment	Proportion of employment
Robot	0.003 (1.423)	-0.087*** (–2.636)	0.004*** (3.205)	–0.050** (–2.155)	0.000** (2.479)	–0.001 (–0.272)
Constant terms	0.856 (0.678)	36.732 (1.114)	0.537 (0.587)	61.528** (2.271)	0.125 (0.823)	9.297* (1.953)
Control variable	Yes	Yes	Yes	Yes	Yes	Yes
Fixed effect of province and year	Yes	Yes	Yes	Yes	Yes	Yes
Sample size	217	217	217	217	217	217

Model (4a) and model (4b) indicated that for each 1 unit increase in industrial robot permeability, the total outstanding employment increased by 0.001 units. The proportion of employment impact was not obvious. Model (4c) and model (4d) indicated that for each 1-unit increase in industrial robot permeability, the impact on the total amount of primary school employment was not obvious. The employment proportion increased by 0.316 percentage points. Model (4e) and model (4f) indicated that for each 1-unit increase in industrial robot permeability, the total employment in junior high schools decreased by 0.042 units. The proportion of employment impact was not obvious. Model (4g) and model (4h) indicated that for each 1-unit increase in industrial robot permeability, the total employment in high school/technical secondary schools decreased by 0.012 units. The proportion of employment impact was not obvious. Model (4i) and model (4j) indicated that for each 1 unit increase in industrial robot permeability, the impact on the total amount of employment in college students was not obvious. The proportion of employment decreased by 0.087 percentage points. Model (4k) and model (4l) indicated that for each 1-unit increase in industrial robot permeability, the total college undergraduate employment increased by 0.004 units. The percentage of employment decreased by 0.050 percentage points. The promotion effect on total employment was higher than the substitution effect of the proportion of employment. Model (4m) and model (4n) indicated that for each 1 unit increase in industrial robot permeability, the total graduate employment increased by 0.0001 units due to decimal retention number issues. The later digits could not be displayed. For each 1-unit increase in industrial robot permeability, the total graduate employment increased by 0.0002332 units. The proportion of employment impact was not obvious. The analysis revealed that the application of industrial robots had a promotion effect on unfinished school and primary school groups, a substitution effect on middle school, high school/technical secondary school, and college specialties, and a promotion effect on college undergraduate and graduate students.

Therefore, the skills of the floating population by education could be divided into three groups: low skills (not school and primary school group), middle skills (high school, high school secondary school. and college specialty group), and high skills (college and graduate group). Industrial robot application had a promoting effect on low- and high-skilled employees, thus validating hypothesis 2.

### Cross-impact of the application of industrial robots on the employment of different skills and employment identities

Previous empirical results showed that the application of industrial robots had a significant effect on the floating population with different skills and employment identities. Furthermore, employees and employers were divided into six groups of low, middle, and high skills to explore the cross-impact. Table 6 reports the impact of industrial robot applications on the employment of the floating population with different skills and employment identities. As a result, industrial robot application had a significant negative impact on low-skilled employees and significant positive effects on high-skilled employers.

**Table 6:** Influence of industrial robot application on the employment of the floating population with different skills and employment identities.

Explain variables	Employees			Employer		
	(5a)	(5b)	(5c)	(5d)	(5e)	(5f)
	Low skills	middle skills	High skills	Low skills	In skills	Highly skilled
Robot	–0.001 (–0.642)	–0.035*** (–4.968)	0.004*** (4.303)	–0.000 (–1.201)	0.002* (1.885)	0.000** (2.476)
Constant terms	0.318 (0.211)	2.303 (0.431)	–0.456 (-0.583)	0.001(0.022)	–0.479 (–1.283)	0.031(0.459)
Control variable	Yes	Yes	Yes	Yes	Yes	Yes
Fixed effect of province and year	Yes	Yes	Yes	Yes	Yes	Yes
Sample size	217	217	217	217	217	217

LM statistic: 35.912***.

C-D Wald F statistic: 1184.739***.

Whether it is total employment or employment proportion, industrial robot application has a significant negative impact on employees, a significant positive impact on employers, and a positive but not significant impact on self-employed workers. Model (3a) and model (3b) showed that for 1 unit of industrial robot permeability, employee employment decreased by 0.320 units, and the employee employment ratio decreased by 0.115 percentage points. Model (3c) and model (3d) showed that for 1 unit of industrial robot permeability, employee employment increased by 0.002 units, and the employer employment ratio increased by 0.047 percentage points. Model (3e) and model (3f) showed that the industrial robot permeability on proprietary worker employment ratio and employment was not significant. Both the absolute and relative quantities indicated that the application of industrial robots had a substitution effect on current employee employment, promoted the employer employment effect, and had no significant impact on the employment of self-employed workers. Hypothesis 2 was verified.

## Conclusion and discussion

In recent years, in research on the domestic and foreign labor markets, the floating population has not had a relatively complete theoretical deduction and empirical analysis. The Chinese empirical evidence shows that the population flow further increased, the floating population labor market was more sensitive, and the floating population skilled workforce with primary and high school education was more vulnerable to the alternative impact of AI. Therefore, this study was based on the labor market of a floating population, the construction of a theoretical model, and the changes in industry employment. It empirically explored the impact of AI on the labor market of the skilled floating population in China and discussed the time trend and industry heterogeneity. The core conclusions were as follows.

First, according to macroeconomic theory models, it can be inferred that as AI technology advances, the automation of skilled labor will progressively replace more jobs, but it will have a relatively minor impact on the substitution of high-skill and low-skill floating population labor. Moreover, this trend is expected to bring about positive effects on productivity and employment in the long run.

Moving on to the empirical analysis of Chinese floating population data, the following hypotheses have been confirmed:

Hypothesis 1: The implementation of industrial robots results in the replacement of existing employment opportunities for the floating population labor force.
Hypothesis 2: The application of industrial robots has the effect of medium-skilled substitution and low- and high-skilled promotion in different skill employment groups. That is, the impact of industrial robots on a heterogeneous skill workforce was bipolar promotion and central substitution.

The development of artificial intelligence has led to changes in the employment structure, with certain skills being superior to others. This change has resulted in a polarization of labor demand for different skill levels. One way in which AI has an impact is through replacing routine daily tasks. These tasks can easily be defined and executed by algorithms, resulting in a reduction in the demand for medium-skilled labor currently engaged in routine tasks. Similar research results have also been presented in other studies [26]. On the other hand, the development of information technology has also created new employment opportunities by integrating information and communication technology into various industries. These new jobs often expand the advantages of highly skilled workers in adaptability and creativity, resulting in a continued increase in demand for highly skilled labor.

First of all, the employment of China’s floating population in China has always been a key issue of the country. Helping vulnerable groups to stabilize employment has always been the macro goal called for by China, and the floating population is one of the vulnerable groups. In this context, we combined with the current development of artificial intelligence to study whether artificial intelligence will squeeze the floating population group? How big is the negative impact? These studies are designed to objectively assess the occupational substitution risk of the floating population to make Chinese policy makers pay attention to this group and give them more practical help. Secondly, the empirical study in this paper shows that artificial intelligence is easier to replace the medium skilled labor force, and more than 60% of the floating population are medium skilled labor force, which has a huge impact on the employment of the floating population. The 2022 China Statistical Yearbook shows that the scale of the floating population has reached more than 30% by the end of 2021, which means that there is one floating population in every three people, showing the transformation characteristics of the floating society. Therefore, the employment problem of the floating population needs to be paid the attention of policy makers, and it is crucial to improve the employment skills of the floating population.

Therefore, in order to balance the current state of the Chinese labor market, this article discusses the three aspects of government, enterprises, and individuals.

For the government, it is necessary to create a fair and open business environment, strengthen market-oriented reforms, clarify the functions of the government and the market, give full play to the advantages of top-level design of the government and market resource allocation, continue to stimulate regional innovation vitality, and improve the efficiency of factor flow including labor force, so as to provide a good business environment for absorbing more labor demand.

For enterprises, it is encouraged to promote transformation and upgrading through deep integration with AI, and at the same time, with the help of local markets, labor, capital, resources and other factors, to create more labor demand for the labor force. Enterprises can further promote economic growth by absorbing highly skilled talents and promoting the overall quality of the labor force, thus forming a virtuous development cycle.

For individuals, migrants should focus on improving their human capital level to cope with the impact of "technological unemployment". Related research indicates that 55%-77% of China’s current labor demand will be replaced by intelligent machines in the future [27]. The rapid development of new technologies associated with the development of artificial intelligence has had a significant impact on employment, especially on the demand for low-education, operational labor, which may bring potential large-scale unemployment risks. For the labor force, it is necessary to actively adapt to the development and innovation of informatization, and promote the compatibility of themselves with information technology in their work positions.

In the future, with the decrease of population and the improvement of population quality, artificial intelligence affect the floating population labor market research can be further refined to the specific industry, occupation, especially the recent rise of odd economy, platform economy, the emergence of new economic form of the floating population employment has a more complex influence. The limitations of this article are: First, the data is only in 2018. Because the database needed in the paper was only investigated in 2018, and it did not continue later, in the future research, we will try to replace the updated data verification results. Second, the impact of artificial intelligence on the floating population is only an industrial industry. In fact, the impact on the service sector may be even greater, and we can continue to do more research along this line of thinking in the future.

## References

[1] Edziah B K, Sun H, Adom P K, Wang F, Agyemang A O. The role of exogenous technological factors and renewable energy in carbon dioxide emission reduction in Sub-Saharan Africa, Renewable Energy, 2022;196:1418–1428. doi: 10.1016/j.renene.2022.06.130

[2] Sun H, Chen T, Wang C N. Spatial impact of digital finance on carbon productivity, Geoscience Frontiers, 2023;101674. doi: 10.1016/j.gsf.2023.101674

[3] Autor DH, Levy F, Murnane Rj. The Skill Content of Recent Technological Change: An Empirical Exploration. Quarterly Journal of Economics. 2003;4: 1279–1333. doi: 10.3386/W8337

[4] Acemoglu D, Restrepo P. The Race between Machine and Man: Implications of Technology for Growth, Factor Shares and Employment. American Economic Review. 2018;6:1488–1542. doi: 10.1257/aer.20160696

[5] Acemoglu D., Restrepo P. Robots and Jobs: Evidence from US Labor Markets. Journal of Political Economy. 2020;6: 2188–2244. doi: 10.2139/SSRN.2940245

[6] Haltiwanger J., Jarmin R.S., Miranda J. Who Creates Jobs? Small vs. Large vs. Young. Review of Economics and Statistics. 2013;2:347–361. doi: 10.1162/REST_a_00288

[7] Acemoglu D, Autor D H. Skills. Tasks and Technologies: Implications for Employment and Earnings. Handbook of Labor Economics. 2011;4: 1043–1171. doi: 10.1016/S0169-7218(11)02410-5

[8] Bresnahan T F, Computerisation. Wage Dispersion. An Analytical Reinterpretation. The Economic Journal, 1999;109(456): 390–415. doi: 10.1111/1468-0297.00442

[9] Autor D H, Katz L F, Kearney M S. The Polarization of the U.S. Labor Market. American Economic Review. 2006;2: 189–194. doi: 10.3386/w15150

[10] Autor D H, Dorn D. The Growth of Low-Skill Service Jobs and the Polarization of the US Labor Market. American Economic Review. 2013;5: 1553–1597. doi: 10.1257/aer.103.5.1553

[11] Frey C B, Osborne M A. The future of employment: How susceptible are jobs to Computerisation? [J]. Technological Forecasting and Social Change, 2017, 114: 254–280. doi: 10.1016/j.techfore.2016.08.019

[12] Lin J. Technological Adaptation, Cities, and New Work [J]. Review of Economics and Statistics, 2011, 93(2): 554–574. doi: 10.1162/REST_a_00079

[13] Goos M, Manning A, Salomons A. Explaining Job Polarization: Routine-Biased Technological Change and Offshoring. American Economic Review. 2014;8: 2509–2526. doi: 10.1257/aer.104.8.2509

[14] Lv S B, Zhang S W. Employment “polarization” in China: an empirical research. China Economic Quarterly. 2015;2: 757–778. doi: 10.1257/aer.104.8.2509

[15] Hao N. An analysis on economic effects of labor force "polarization"——based on two perspectives of economic growth and income inequality. East China Economic Management. 2017;2: 118–125. doi: 10.3969/j.issn.1007-5097.2017.02.016

[16] Qu X B, Cheng J. Changes of employment structure in China: Upgrading or polarization? Studies in Labor Economics. 2015;1: 119–145. CNKI:SUN:LDJJ.0.2015-01-006.

[17] Li H B, Guo J X, Zhai R R. Does outward foreign direct investment affect job polarization in the China’s labor market? Journal of Finance and Economics. 2017;6: 28–39. doi: 10.16538/j.cnki.jfe.2017.06.003

[18] Sun Z, Hou Y L. How does industrial intelligence reshape the employment structure of Chinese labor force. China Industrial Economics. 2019;5: 61–79. doi: 10.19581/j.cnki.ciejournal.2019.05.004

[19] Li S Q, Wang H C. The impact of artificial intelligence on the manufacturing labor force in the context of agieing: Evidence from China. Science of Science and Management of S.& T. 2021;7: 3–17.

[20] Dong X S, Xiao X. Does the change of demographic dividend affect China’s industrialization? China Population, Resources and Environment. 2016;9: 20–27.

[21] Bartik T J. Who Benefits from State and Local Economic Development Policies? Kalamazoo, M.: W.E. Upjohn Institute. doi: 10.17848/9780585223940

[22] Acemoglu D, Akcigit U, Kerr W. Networks and the Macroeconomy: An Empirical Exploration. NBER Macroeconomics Annual. 2016; 1: 273–335. doi: 10.1086/685961

[23] Cheng H, Jia R X, Li D D, Li H B. The Rise of Robots in China. Journal of Economic Perspectives. 2019;3: 71–88. doi: 10.1257/jep.33.2.71

[24] Han M C, Zhao Y F. The employment effect of industrial robots in Chinese manufacturing. Journal of industrial technological economics. 2019;11: 3–12.

[25] Wei X H, Zhang P K, Du Y H. How robots reshape the urban labor market: From a perspective of migrants’ job tasks. Economic perspectives. 2020;10: 92–109.

[26] Alexopoulos M, Cohen J. The Medium Is the Measure: Technical Change and Employment, 1909–1949. Review of Economics and Statistics, 2016, 98(4): 792–810. doi: 10.1162/REST_a_00588

[27] Acemoglu D, Restrepo P. Robots and Jobs: Evidence from US Labor Markets. Journal of Political Economy, 2020;128(6): 2188–2244. doi: 10.1086/705716

